# An autophagy-inducing stapled peptide induces mitochondria dysfunction and triggers autotic cell death in triple-negative breast cancer

**DOI:** 10.1038/s41420-023-01600-0

**Published:** 2023-08-19

**Authors:** Xiaozhe Zhang, Gao Shan, Na Li, Jingyi Chen, Changyang Ji, Xiaoxiao Li, Liwen Jiang, Terence Kin Wah Lee, Vincent W. Keng, Yanxiang Zhao

**Affiliations:** 1 Department of Applied Biology and Chemical Technology, State Key Laboratory of Chemical Biology and Drug Discovery, The Hong Kong Polytechnic University, Hung Hom, Kowloon, 999077 Hong Kong, P. R. China; 2 The Hong Kong Polytechnic University Shenzhen Research Institute, 518057 Shenzhen, P. R. China; 3 School of Life Sciences, Centre for Cell & Developmental Biology, State Key Laboratory of Agrobiotechnology, The Chinese University of Hong Kong, Shatin, New Territories, Hong Kong, China

**Keywords:** Macroautophagy, Peptides, Macroautophagy

## Abstract

Autophagy is a lysosome-dependent bulk degradation process essential for cell viability but excessive autophagy leads to a unique form of cell death termed autosis. Triple-negative breast cancer (TNBC) is a highly aggressive subtype of breast cancer with notable defect in its autophagy process. In previous studies, we developed stapled peptides that specifically targeted the essential autophagy protein Beclin 1 to induce autophagy and promote endolysosomal trafficking. Here we show that one lead peptide Tat-SP4 induced mild increase of autophagy in TNBC cells but showed potent anti-proliferative effect that could not be rescued by inhibitors of programmed cell death pathways. The cell death induced by Tat-SP4 showed typical features of autosis including sustained adherence to the substrate surface, rupture of plasma membrane and effective rescue by digoxin, a cardioglycoside that blocks the Na^+^/K^+^ ATPase. Tat-SP4 also induced prominent mitochondria dysfunction including loss of mitochondria membrane potential, elevated mitochondria reactive oxygen species and reduced oxidative phosphorylation. The anti-proliferative effect of Tat-SP4 was confirmed in a TNBC xenograft model. Our study uncovers three notable aspects of autosis. Firstly, autosis can be triggered by moderate increase in autophagy if such increase exceeds the endogenous capacity of the host cells. Secondly, mitochondria may play an essential role in autosis with dysregulated autophagy leading to mitochondria dysfunction to trigger autosis. Lastly, intrinsic autophagy deficiency and quiescent mitochondria bioenergetic profile likely render TNBC cells particularly susceptible to autosis. Our designed peptides like Tat-SP4 may serve as potential therapeutic candidates against TNBC by targeting this vulnerability.

## Introduction

Triple-negative breast cancer (TNBC) is a highly aggressive subtype of breast cancer with frequent relapse or metastasis after first-line treatment by surgery and chemotherapy [1–3]. TNBC lacks expression or amplification of three molecular markers, i.e., estrogen receptor (ER), progesterone receptor (PR) and human epidermal growth factor receptor 2 (HER2) [1–3]. As a result, TNBC does not respond to hormonal therapies or HER2-targeting drugs that are highly effective for ER^+^/PR^+^/HER2^+^ breast cancer. A few targeted therapeutics have been approved recently with moderate survival benefit and limited response rate. For example, PARP inhibitors including olaparib and talazoparib have been approved for patients with advanced or metastatic TNBC and carrying germline BRCA mutation, which has a prevalence rate of 10–30% [4, 5]. Immune checkpoint inhibitors including atezolizumab and Pembrolizumab used in combination with chemodrugs showed significant survival benefit but the overall response rate (ORR) was ~20% in PD-L1^+^ patients and only ~5% for all patients regardless of PD-L1 expression [6–8]. Sacituzumab Govitecan, an antibody–drug conjugate consisting of an anti-Trop-3 antibody and a topoisomerase inhibitor, was recently approved as a third-line treatment for metastatic TNBC after achieving the impressive result of extending the overall survival by more than 5 months and ORR of ~35% [9, 10]. Nonetheless, novel therapeutic targets and modalities are urgently needed to offer more effective treatments for the broader patient population of TNBC.

Autophagy is an evolutionarily conserved lysosome-dependent bulk degradation process that sequesters cytosolic content such as proteins and organelles into double-membraned autophagosomes and delivers them to lysosomes for degradation and recycling [11–15]. Autophagy has an intimate and yet complex relationship with cancer, acting like a double-edged sword in context-dependent manner [11, 16–19]. At the early stage in pre-malignant cells, autophagic response is regarded as an anti-tumor mechanism to preserve cellular integrity and inhibit malignant transformation [16, 17, 19]. At later stage of tumorigenesis, autophagy is believed to play a pro-tumor role to sustain the growth and progression of established tumors within the metabolically stressed microenvironment [16, 17, 19]. Thus it has been a challenge to target the autophagy process effectively to inhibit proliferation of cancer cells.

Beclin 1 is an essential mammalian autophagy gene and a haploinsufficient tumor suppressor [20–22]. It is mapped to the tumor susceptibility chromosomal locus 17q21 and monoallelically deleted in 40–75% of cases of human sporadic breast, ovarian, and prostate cancer [20, 21]. Mouse studies have confirmed the tumor suppressor role of Beclin 1 as heterozygous knockout of Beclin 1 leads to higher rate of spontaneous malignancies such as lymphomas and lung or hepatocellular carcinomas [21, 23]. Beclin 1 has close relationship to TNBC as bioinformatic analysis has revealed that low mRNA level of Beclin 1 is more commonly detected in TNBC than in other breast cancer subtypes [24]. This low expression level of Beclin 1 is also strongly associated with advanced tumor grade and poor prognosis [24]. Furthermore, there is evidence that autophagic activity in TNBC is impaired and this deficiency promotes resistance to immune checkpoint inhibitors like anti-PD-1/PD-L1 antibodies [25]. We reason that the low expression level of Beclin 1 in TNBC, which likely leads to autophagy deficiency, may be a targetable vulnerability.

Beclin 1 is a core member of the Class III phosphatidylinositol-3-kinase (PI3KC3) complex. By recruiting positive regulators like Atg14L or UVRAG, Beclin 1 facilitates the formation of Atg14L/UVRAG-containing PI3KC3 complexes with significantly elevated kinase activity [26–29]. In our previous studies, we designed and characterized a series of Beclin 1-targeting peptides with hydrocarbon staples added to stabilize their alpha helical structure so that they would bind to the coiled coil domain of Beclin 1 to reduce its self-dimerization and to promote its interaction with Atg14L and UVRAG [30, 31]. In non-small-cell lung cancer (NSCLC) cells like A549 and H1975, these designed peptides promoted autophagy and enhanced endolysosomal degradation of the over-expressed cell surface oncogenic receptor epidermal growth factor receptor (EGFR), two processes that are Beclin 1-dependent [30]. These designed peptides also showed similar effect in HER2^+^ breast cancer cells and exerted potent anti-proliferative effect by inducing necrotic cell death but not apoptosis [31].

Here we report that a lead candidate Tat-SP4 selected from these Beclin 1-targeting stapled peptides induced autophagy and promoted endolysosomal degradation of EGFR in multiple TNBC cell lines. Intriguingly, Tat-SP4 induced necrotic cell death with notable features of autosis, a form of cell death related to excessive autophagy. Tat-SP4 also triggered mitochondria dysfunction with loss of mitochondrial membrane potential, elevated level of reactive oxygen species (ROS) and reduced oxidative phosphorylation (OXPHOS) activity. Tat-SP4 also effectively inhibited tumor growth in a xenograft model of TNBC. These results validate Beclin 1-mediated autophagy as a targetable vulnerability for TNBC with our designed peptides as potential therapeutic candidates.

## Results

### A Beclin 1-targeting stapled peptide Tat-SP4 leads to moderate increase of autophagy in TNBC cells

**Fig. 1:**
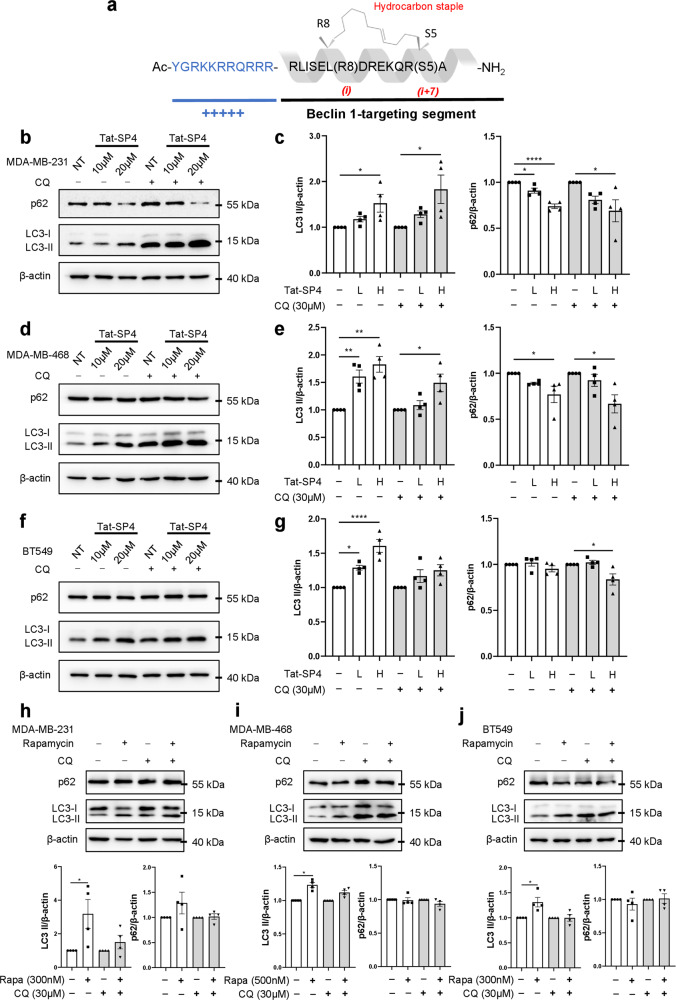
Tat-SP4 induced autophagy in TNBC cell lines. **a** Sequence and chemical structure of Tat-SP4. A cell-penetrating Tat sequence (colored in blue) is added to the N-terminal of Tat-SP4. C-terminal is the Beclin-1 targeting segment. The α-helical structure is stabilized by hydrocarbon staple. **b**, **d**, **f** Western blot to assess the p62 level and LC3 lipidation profile in **b** MDA-MB-231, **d** MDA-MB-468, and **f** BT549 cell lines after treatment with 10 and 20 μM of Tat-SP4 for 3 h, in the presence or absence of 30 μM CQ. **c**, **e**, **g** Quantification of p62 levels and LC3 lipidation profiles from the western blot data. The levels of LC3-II and p62 were normalized to the β-actin level. L: low concentration (10 μM); H: high concentration (20 μM). **h**, **i**, **j** Western blot to assess the p62 level and LC3 lipidation profile in **h** MDA-MB-231, **i** MDA-MB-468, and **j** BT549 cell lines after treatment with the indicated concentration of rapamycin for 3 h. Data are presented as mean ± SEM (*n* = 4); *P < 0.05, **P < 0.01, ****P < 0.0001, and individual points represent independent wells of cultured cells.

Our previous studies developed two batches of Beclin 1-targeting stapled peptides [30, 31]. Both batches have the same overall scaffold with the N-terminal Tat sequence of YGRKKRRQRRR to facilitate cell membrane penetration and the C-terminal portion to specifically target the Beclin 1 coiled coil domain (Fig. 1a) [30, 31]. A hydrocarbon staple of 13-carbon length was installed at residues *i* and *i* + *7* to maintain its alpha helical structure, a feature critical for Beclin 1 binding [30]. The second batch yielded lead candidates with ~10–30× stronger binding affinity to Beclin 1 because their hydrocarbon staple was placed closer to the Beclin 1-peptide interface [31]. However, lead candidates from the second batch showed poorer solubility than the first batch, which rendered them unsuitable for animal studies [31]. Based on a balanced consideration of the binding affinity and physicochemical properties of the peptide, we decided to use the lead candidate Tat-SP4 from the first batch to investigate its anti-proliferative efficacy in TNBC.

TNBC is a heterogeneous disease involving cell types of diverse genetic background, distinct morphological features and varied clinical behavior [1–3]. Classification of TNBC has been attempted using histopathological evaluation, gene expression profiling or genomic analysis but a unified model to inform diagnosis and clinical treatment has not been established [1–3]. One prevailing model based on gene expression profiling analysis of clinical samples has led to classification of four distinct subtypes including basal-like 1 and 2 (BL1 and BL2), mesenchymal (M), and the luminal androgen receptor (LAR) [32–35]. Here we have chosen three TNBC cell lines to represent two distinct subtypes including MDA-MB-231 and BT549 for the M subtype and MDA-MB-468 for the BL1 subtype.

The impact of Tat-SP4 on cellular autophagic activity of these three TNBC cell lines was examined by measuring the levels of two autophagy markers including LC3-II, a lipidated form of cytosolic protein LC3 that become enriched on autophagosomes, and p62, an autophagy receptor protein [30]. Our results show that treatment of the three TNBC cell lines with Tat-SP4 at 10 and 20 μM for 3 h induced varied responses. In MDA-MB-231 cells, Tat-SP4 induced autophagy in dosage-dependent manner with increase in the LC3-II level and concomitant decrease in p62 level (Fig. 1b, c). Addition of the chloroquine (CQ), a lysosomal inhibitor, led to overall increase of the LC3-II level but did not affect the changes in LC3-II and p62 induced by Tat-SP4 (Fig. 1b, c). Similar affect was observed in MDA-MB-468 cells, with Tat-SP4 inducing noticeable increase in the LC3-II level with concomitant decrease of the p62 level (Fig. 1d, e). For BT549 cells, Tat-SP4 induced noticeable albeit small increase in the LC3-II level but the p62 level remained steady (Fig. 1f, g). Notably, BT549 showed low level of LC3-I relative to LC3-II, thus suggesting limited spare capacity in terms of further enhancing the conversion from LC3-I to LC3-II upon autophagy induction. Thus Tat-SP4 induced moderate increase of autophagic response in MDA-MB-231 and MDA-MB-468 in dosage-dependent manner while BT549 showed only subdued response due to its basal autophagy state with limited spare capacity for further enhancement. To further evaluate the basal autophagy activity in the three TNBC cell lines, we assessed their response to rapamycin, an autophagy inducer. Our data shows that rapamycin induced the most robust autophagic response in MDA-MD-231 as its LC3-II level showed the largest increase (Fig. 1h). In comparison, the LC3-II level in MDA-MB-468 (Fig. 1i) and BT549 (Fig. 1j) showed much smaller increase. This data supports our statement that BT549 has lower spare capacity as compared to MDA-MB-231.

### Tat-SP4 significantly enhances endolysosomal degradation of EGFR in TNBC cells

**Fig. 2:**
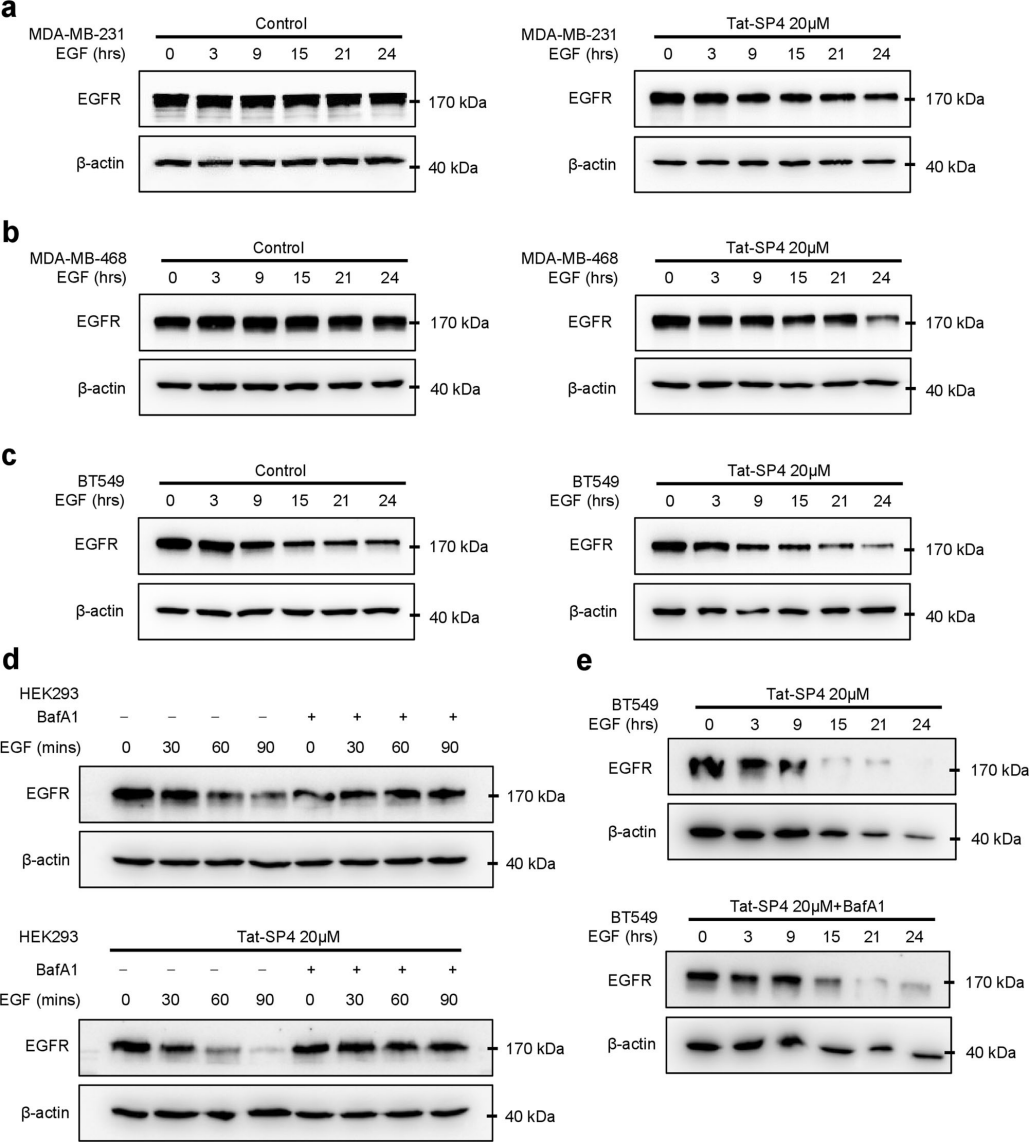
Tat-SP4 promoted endolysosomal degradation in TNBC cells. **a**–**c** Western blot to assess the EGFR levels in **a** MDA-MB-231, **b** MDA-MB-468, **c** BT549 cell lines. Cells were starved overnight and were treated with 200 ng/ml EGF together with vehicle control or 20 μM Tat-SP4 for the indicated times. **d**, **e** Western blot to assess the EGFR levels in **d** HEK293 and **e** BT549 cell lines. Cells were starved overnight and were treated with 200 ng/ml EGF with or without 20 μM Tat-SP4 and 100 nM Baf A1 for the indicated times, with or without the lysosome inhibitor Baf A1 (100 nM).

Our previous studies showed that Tat-SP4 promoted endolysosomal degradation of EGFR and HER2, which are oncogenic drivers for non-small cell lung cancer (NSCLC) and HER2+ breast cancer subtype respectively [30]. While TNBC shows low or no expression of HER2, EGFR is frequently overexpressed [3]. To assess whether Tat-SP4 exerts similar effect in TNBC, the ligand-induced endolysosomal degradation of EGFR was tracked by western blotting in the three TNBC cell lines. In both MDA-MB-231 and MDA-MB-468 cells, treatment by the agonist EGF did not trigger any noticeable endolysosomal degradation activity as the level of EGFR was steady over a period of 24 h (Fig. 2a, b). In contrast, BT549 cells were highly susceptible to EGF treatment with EGFR degraded by ~50% after 24 h (Fig. 2c). Tat-SP4 treatment at 20 μM led to significant enhancement of EGFR degradation in MDA-MB-231 and MDA-MB-468, with EGFR level decreased by ~50 and ~70%, respectively (Fig. 2a, b). Similar effect was observed in BT549 cells treated with Tat-SP4 as well, showing ~10% additional EGFR degradation on top of its intrinsic high turnover rate (Fig. 2c). These data confirm that Tat-SP4 significantly enhances endolysosomal degradation of EGFR in TNBC.

To further confirm that Tat-SP4 enhanced the endolysosomal pathway to promote EGFR degradation, we assessed whether this effect would be blocked by Baf A1, a lysosomal inhibitor. We first tested the impact of Baf A1 in HEK293 cells. Our data shows that, in absence of Tat-SP4, EGF treatment induced notable degradation of EGFR. Such degradation was effectively blocked by Baf A1, thus validating the involvement of endolysosomal pathway in EGFR degradation (Fig. 2d). Furthermore, Tat-SP4 significantly enhanced EGF-induced EGFR degradation but Baf A1 totally blocked such enhancement (Fig. 2d). We also did such experiments using BT549 cells and obtained similar results (Fig. 2e). MDA-MB-231 and MDA-MB-468 cells were not tested because neither showed obvious EGF-induced EGFR degradation. Overall, our data confirms that the enhanced EGFR degradation after Tat-SP4 treatment depends on the endolysosomal pathway.

### Tat-SP4 exerts potent anti-proliferative effect on TNBC cell lines

**Fig. 3:**
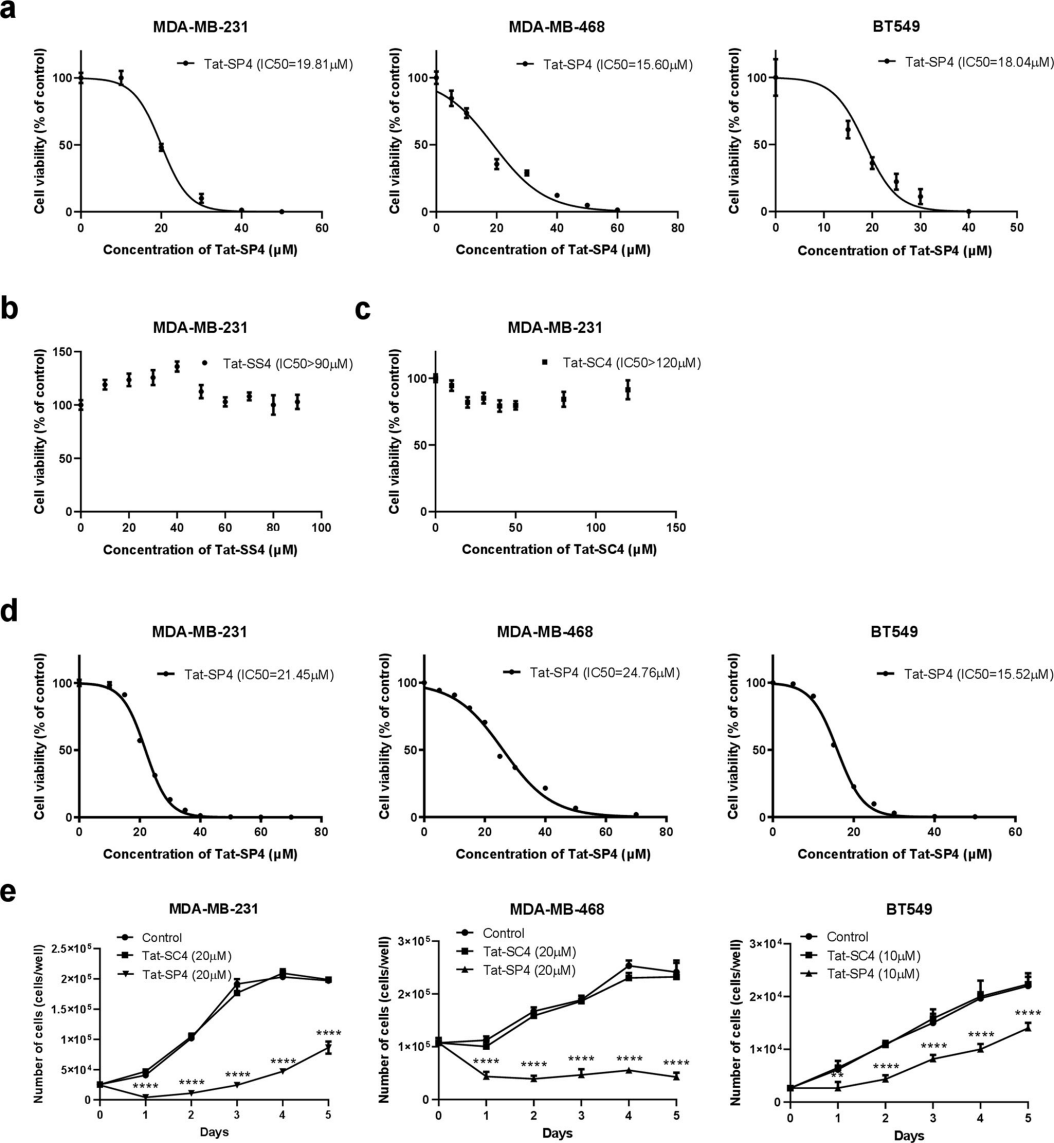
Tat-SP4 exerts potent anti-proliferative effect on TNBC cell lines. **a** Trypan blue exclusion assay to assess the cytotoxicity IC50 of Tat-SP4 in MDA-MB-231, MDA-MB-468 and BT549 cell lines. Cells were treated with various concentrations of the Tat-SP4 for 24 h. Cell numbers were manually counted by the trypan blue dye exclusion method using a hemocytometer. **b**, **c** Cytotoxicity of control peptide, Tat-SS4 and Tat-SC4, in MDA-MB-231 cell line by trypan blue exclusion assay. **d** Cytotoxicity of Tat-SP4 in MDA-MB-231, MDA-MB-468 and BT549 cell lines measured by the CellTiter-Glo assay. **e** Cell proliferation assay was performed over a time span of 5 days in MDA-MB-231, MDA-MB-468 and BT549 cell lines after treatment with the indicated concentrations of Tat-SP4. Cells were treated with Tat-SP4 at day 0 and the cell number was calculated every 24 h. Data are presented as mean ± SEM from three independent experiments; **P < 0.01, ****P < 0.0001.

To evaluate the anti-proliferative potency of Tat-SP4, we used trypan blue exclusion assay to measure its IC_50_ values on the TNBC cell lines. Our results show that Tat-SP4 exerted potent anti-proliferative effect on all three cell lines with IC_50_ of ~15–18 μM (Fig. 3a). However, no such effect was observed for two control peptides, including Tat-SC4 that is an unmodified peptide with scrambled sequence and Tat-SS4 that has the same sequence as Tat-SC4 but contains a hydrocarbon staple like Tat-SP4 (Fig. 3b, c and Table 1). We also obtained similar IC_50_ values using CellTiter-Glo, a different assay for measuring cell viability (Fig. 3d). A five-day proliferation assay confirmed that Tat-SP4 reduced the proliferation of MDA-MB-231 cells by ~50% at 20 μM while the control peptide Tat-SC4 showed no such effect (Fig. 3e). Tat-SP4 also completely abolished the proliferation of MDA-MB-468 cells at 20 μM for 5 days while it reduced the proliferation of BT549 cells by ~40% at the lower dosage of 10 μM (Fig. 3e). These results confirm that Tat-SP4 potently inhibits the proliferation of TNBC cells across diverse genetic background.

**Table 1:** The sequence of peptides.

Peptide	Sequence
Tat-SP4	Ac-**YGRKKRRQRRR**-RLISEL(R8)DREKQR(S5)A-NH_2_
Tat-SS4	Ac-**YGRKKRRQRRR**-RALRIQ(R8)SKEELR(S5)D-NH_2_
Tat-SC4	Ac-**YGRKKRRQRRR**-RALRIQSKEELRD-NH_2_

^*^The sequence highlighted in bold indicates the Tat sequence. The Tat sequence is added to facilitate cell penetration.

### Tat-SP4 triggers necrotic cell death that is only rescued by an inhibitor of autosis

**Fig. 4:**
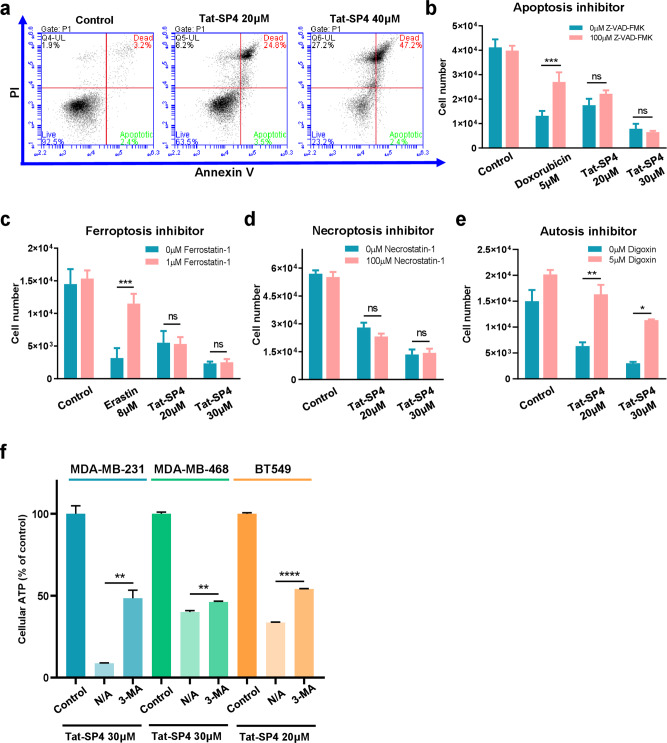
Tat-SP4-induced cell death is rescued by an inhibitor of autosis. **a** Flow cytometry analysis with Annexin V FITC-PI staining was performed in MDA-MB-231 cells after treatment of the indicated peptides for 24 h. The percentage of dead cells (PI positive) in Tat-SP4 treatment groups were significantly increased compared with that of control, without notable changes in the population of apoptotic cells. **b**, **c** Trypan blue exclusion method to assess the viability of MDA-MB-231 cells upon treatment with indicated concentrations of Tat-SP4 or **b** 5 μM doxorubicin or **c** 8 μM Erastin, in the presence or absence of **b** 100 μM Z-VAD-FMK or 1 μM Ferrostatin-1 for 24 h. Z-VAD-FMK: a pan-caspase inhibitor; Erastin: ferroptosis inducer; Ferrostatin-1: a selective ferroptosis inhibitor. **d**, **e** Trypan blue exclusion method to assess the viability of MDA-MB-231 cells with treatment of indicated concentrations of Tat-SP4 in the presence or absence of **d** 100 μM Necrostatin-1 or **e** 5 μM digoxin for **d** 24 h or **e** 5 h. **f** Viability of MDA-MB-231, MDA-MB-468 and BT549 cells was assessed by CellTiter-Glo assay after treatment with the indicated concentrations of Tat-SP4 in the presence or absence of 10 mM 3-MA. Data are presented as mean ± SEM from three independent experiments; *P < 0.05, **P < 0.01, ***P < 0.001, ****P < 0.0001.

Our previous study reported that Beclin 1-targeting stapled peptides induced necrotic cell death but not apoptosis in HER2+ breast cancer cells [31]. We set out to investigate if Tat-SP4 caused similar effect on TNBC cells to inhibit their proliferation. Flow cytometry experiments with annexin V and propidium iodide (PI) staining revealed that Tat-SP4 induced necrotic cell death but not apoptosis in dosage-dependent manner (Fig. 4a). Treatment of Tat-SP4 at 20 μM led to ~24.8% dead cells that stained positive for both annexin V and PI, thus indicative of compromised plasma membrane and necrotic cell death (Fig. 4a). Higher dosage of Tat-SP4 at 40 μM led to 47.2% necrotic cell death while the number of apoptotic cells that stained positive only for annexin V remained at ~3-5% for both the control and after Tat-SP4 treatment at two different dosages (Fig. 4a). Thus Tat-SP4 induced necrotic cell death in TNBC cells similar to HER2^+^ breast cancer cells.

Besides apoptosis, there are other pathways of regulated cell death such as necroptosis, ferroptosis and pyroptosis that also show necrotic features at late stage including compromised cell membrane [36]. To assess whether these pathways were involved, we tried to test whether inhibitors of these pathways could rescue the necrotic cell death induced by Tat-SP4 in TNBC cells. Our data shows that Z-VAD-FMK, a pan-caspase inhibitor, readily rescued the apoptotic cell death caused by doxorubicin but showed little rescue effect for Tat-SP4 (Fig. 4b). Similar results were obtained for necrostatin-1, an inhibitor of necroptosis, and ferrostatin-1, an inhibitor of ferroptosis (Fig. 4c, d). Thus, the necrotic cell death induced by Tat-SP4 did not involve any of these pathways of programmed cell death.

Autosis is a particular type of cell death caused by excessive autophagic activity [37]. It can be triggered by a few stress factors if applied excessively, such as prolonged starvation, hypoxia–ischemia and viral infection [38–42]. It can also be induced by high dosage of an autophagy-inducing peptide termed Tat-Beclin 1 that contains an N-terminal Tat sequence and a C-terminal Beclin 1-derived segment responsible for binding to the Nef protein of human immunodeficiency virus (HIV) [38]. The pathway of autosis is regulated by the Na^+^/K^+^ ATPase, an ion channel critical for maintaining Na^+^ and K^+^ gradients across the plasma membrane [38]. Conversely, inhibitors of the Na^+^/K^+^ ATPase, such as cardiac glycosides digoxin and aubain, have been found to rescue autosis [38–40]. Interestingly, digoxin readily rescued the necrotic cell death induced by Tat-SP4 (Fig. 4e). 3-MA, an early-stage autophagy inhibitor, also blocked Tat-SP4-induced cell death in TNBC cells (Fig. 4f). Thus Tat-SP4 may induce the autophagy-dependent autosis process in TNBC cells similar to Tat-Beclin 1 [38].

### Cell death triggered by Tat-SP4 shows morphological features of autosis and mitochondria dysfunction

**Fig. 5:**
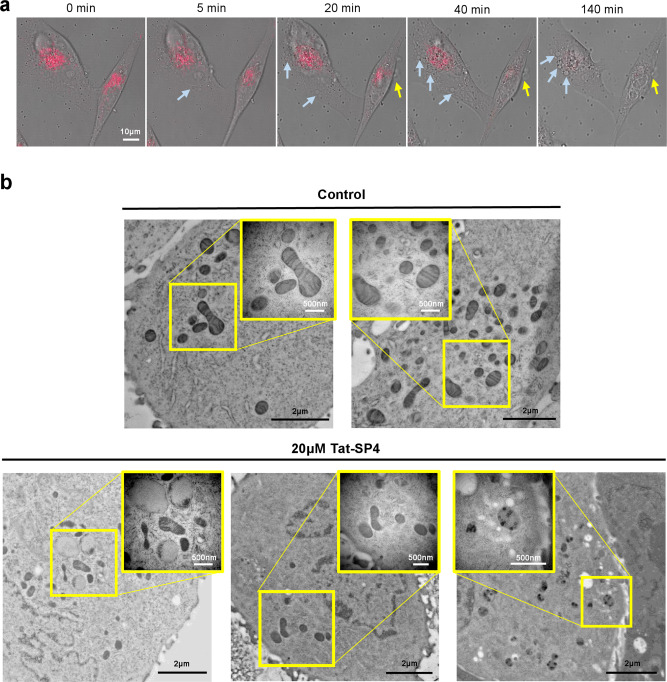
Tat-SP4-induced cell death shows morphological features of autosis and unique mitochondria damage. **a** MDA-MB-231 cells stained with TMRM for 30 min followed by 30 μM Tat-SP4 treatment were subjected to confocal imaging for 3 h (Movie S1). The representative images were selected to present the mitochondrial membrane potential and cellular morphology change. The blue arrows indicate the excessive vacuolization; the yellow arrows indicate the leakage of cytosolic contents. Scale bar: 10 μm. **b** Ultrastructural analysis of MDA-MB-231 with or without 1-h treatment of 20 μM Tat-SP4 by TEM. Scale bar is 2 μm in zoom-out version and 500 nm in zoom-in version, respectively.

Autosis shows distinctive morphological features compared to other cell death pathways. Cells undergoing autosis in vitro remain attached to the culture dish while apoptotic and necroptotic cells usually float [38]. Autotic cells also show excessive vacuolization, expansion of perinuclear space and eventual rupture of plasma membrane [38]. To assess whether Tat-SP4 induced cell death showed any of these morphological features, we first performed live-cell imaging of Tat-SP4 treated MDA-MB-231 cells (Fig. 5a and Movie S1). Within 20 min after treatment by 30 μM Tat-SP4, MDA-MB-231 cells began to show gradual loss of mitochondrial membrane potential as monitored by the TMRM fluorescent dye. Cell motility also decreased and a few large vacuoles appeared in the cytoplasm although cell morphology remained largely unaffected. As loss of the TMRM signal reached certain threshold (estimated to be ~50%), cells underwent necrotic death as manifested by abrupt but localized rupture of plasma membrane and leakage of cytoplasmic contents while still attached to the cover slip (Fig. 5a and Movie S1). This process of Tat-SP4 triggered cell death does share similar features with the autosis process reported in earlier studies such as the rupture of plasma membrane, leakage of cytosolic content and increased substrate adhesion [38]. However, certain features of the autosis process induced by the Tat-Beclin 1 peptide and starvation, such as excessive vacuolization and rapid shrinkage of the nucleus were not observed [38].

To further analyze the cell death process induced by Tat-SP4, we carried out ultrastructural analysis by transmission electron microscopy (TEM). MDA-MB-231 cells were treated with 20 μM Tat-SP4 for 1 h and then subject to fixation and analysis by TEM. Compared to untreated cells, Tat-SP4 treatment caused significant damage to mitochondria, with many becoming electron dense and losing their cristae structure (Fig. 5b). Some mitochondria even contained multiple electron dense precipitates, likely indicative of their total demise (Fig. 5b). Other organelles such as the nucleus and ER appeared normal and no excessive number of autophagosomes were observed (Fig. 5b). While HeLa cells treated with Tat-Beclin 1 peptide showed ultrastructural changes involving multiple organelles before the final step of cell death [38], our TEM analysis revealed that ultrastructural changes in MDA-MB-231 cells treated with Tat-SP4 were confined to mitochondria.

### Tat-SP4 impairs mitochondria function and oxidative phosphorylation activity

**Fig. 6:**
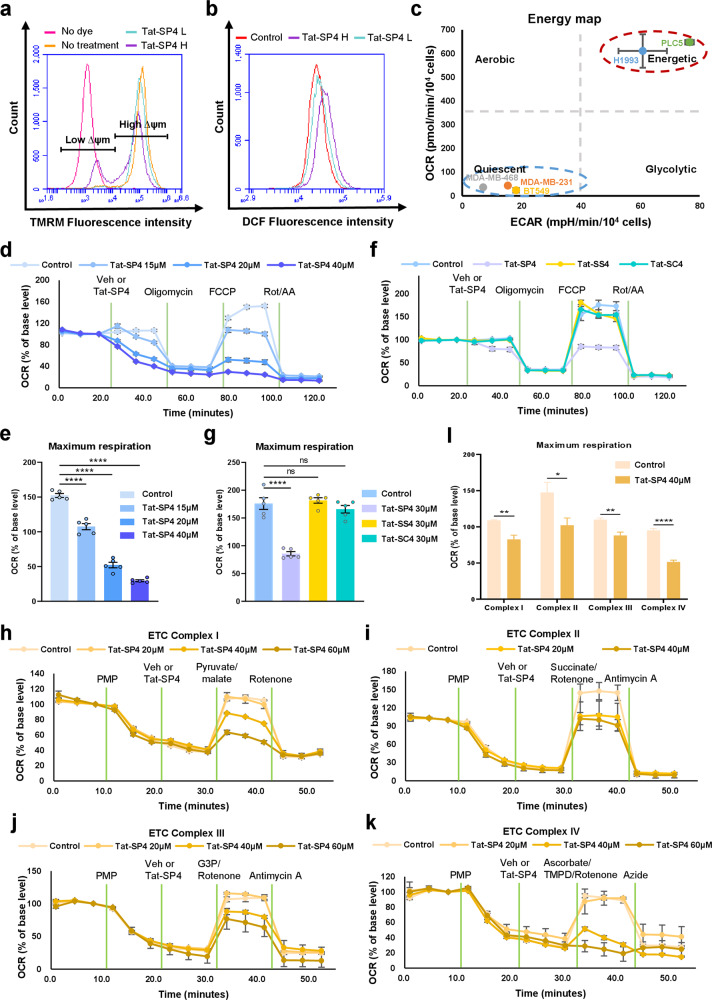
Tat-SP4 impairs mitochondria membrane potential and oxidative phosphorylation activity. **a** Representative flow cytometry plot of MDA-MB-231 cells with the treatment of indicated concentrations of Tat-SP4 for 30 min. Cells were stained with TMRM for 30 min and analyzed in flow cytometry. L: low concentration (20 μM); H: high concentration (40 μM). **b** Representative flow cytometry plot of the changes in fluorescence intensity of DCF, an indicator dye of cellular ROS, under the treatment of indicated concentrations of Tat-SP4 for 30 min. Fluorescence intensity is directly proportional to the amount of ROS species in the cell. L: low concentration (20 μM); H: high concentration (40 μM). **c** Energy map was performed by seahorse analysis. ECAR and OCR data were plotted to reveal the basal metabolic profile of each cell line. Compared to other cancer cell lines (PLC5, H1993), TNBC cell lines (MDA-MB-231, MDA-MB-468, BT549) are more quiescent. Data are presented as mean ± SEM (*n* = 5). **d**, **f** The change of OCR induced by **d** different concentrations (15 μM, 20 μM, 40 μM) of Tat-SP4 or **f** control peptides (Tat-SS4, Tat-SC4) was measured by Agilent Seahorse Analyzer using Mito Stress Test. **d** MDA-MB-231 cells or **f** MDA-MB-468 cells were treated with indicated concentrations of the peptides or vehicle (control) followed by sequential injection of 1 μM oligomycin, 1 μM FCCP, and 0.5 μM rotenone and antimycin A. Each data point represented the relative OCR compared to the base level. Data are presented as mean ± SEM (*n* = 5). **e**, **g** Quantification of maximum respiration change in (**d**, **f**), ****P < 0.0001. **h**–**k** The effect of Tat-SP4 on each ETC complex was measured in permeabilized MDA-MB-231 cells. 1 nM PMP was used to permeabilize cells and followed by the treatment of vehicle or indicated concentrations of Tat-SP4. To stimulate individual complex, **h** 10 mM pyruvate/1 mM malate (Complex I), **i** 10 mM succinate/2 μM rotenone (Complex II), **j** 10 mM glycerol-3-phosphate/2 μM rotenone, or **k** 10 mM ascorbate and 100 μM TMPD + 2 μM antimycin A (Complex IV) was injected, respectively. Each data point represented the relative OCR compared to the base level. Data are presented as mean ± SEM (*n* = 5). **l** Quantification of OCR change induced by Tat-SP4 in each ETC complex (**h**–**k**), *P < 0.05, **P < 0.01, ****P < 0.0001.

Mitochondria play pivotal roles in multiple cell death programs [36]. Additionally, autophagy and mitochondria function are known to be closely interconnected, such that disruption of one process likely impacts the other [11–15]. As our live-cell imaging studies and TEM analysis suggested that Tat-SP4 caused mitochondria damage, we then carried out further analysis to assess whether Tat-SP4 would impair mitochondria function. Flow cytometry analysis of MDA-MB-231 cells showed that Tat-SP4 reduced mitochondria membrane potential (Δψ) and led to higher level of cellular ROS in dosage-dependent manner (Fig. 6a, b). The impact of Tat-SP4 on cellular bioenergetics was assessed by the Agilent Seahorse XF Analyzer, with the mitochondria-mediated oxidative phosphorylation activity measured by the oxygen consumption rate (OCR) and the glycolysis process measured by extracellular acidification rate (ECAR). In comparison to the hepatocellular carcinoma cell line PLC5 and the non-small cell lung cancer cell line H1993, all three TNBC cells were notably more quiescent with lower OCR and ECAR levels (Fig. 6c). Previous studies have also reported similar bioenergetics profile for TNBC cells [43]. Tat-SP4 significantly reduced the maximal OCR in dosage-dependent manner but only had moderate effect on the basal OCR (Fig. 6d, e). In comparison, control peptides Tat-SS4 and Tat-SC4 showed no effect on maximal OCR (Fig. 6f, g).

As there are four complexes (I–IV) in the mitochondria electron transfer chain that work together to determine the overall OXPHOS activity, we investigated whether Tat-SP4 specifically blocked any particular complex. MDA-MB-231 cells were permeabilized and treated with substrates specific for Complexes I–IV in the absence or presence of Tat-SP4. Our data shows that Tat-SP4 affected the maximal OCR of all complexes I–IV in dosage-dependent manner (Fig. 6h–l). All these data suggest that Tat-SP4 likely impaired mitochondria function by reducing mitochondria membrane potential (Δψ), thus leading to redox stress and decrease in OXPHOS activity.

### Tat-SP4 inhibits tumor growth in a TNBC xenograft model

**Fig. 7:**
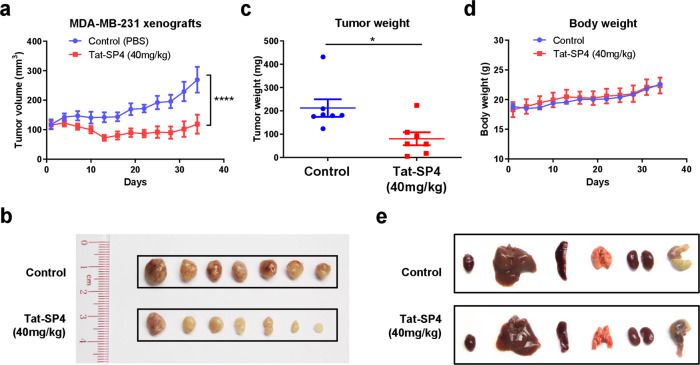
Tat-SP4 inhibits TNBC tumor growth in a xenograft model. **a** MDA-MB-231 cells were subcutaneously injected into nude mice in a number of 5 × 10^6^. The administration of 40 mg/kg Tat-SP4 or PBS (control) was started when the tumor volume reached 100 mm^3^. The tumor volume was recorded every 3 days from the initial treatment to tumor harvest (day 34). The tumor volume was defined as width^2^ × length/2. Data are presented as mean ± SEM; *n* = 7; ****P < 0.0001. **b** Picture of the dissected MDA-MB-231 tumor tissues from each mouse. **c** The tumor weight was measured after collection on day 34. Data are presented as mean ± SEM; *n* = 7; *P < 0.05. **d** The development of body weight in mice after receiving the treatment of 40 mg/kg Tat-SP4 or PBS. Data are presented as mean ± SEM; *n* = 7. **e** The representative pictures of harvested vital organs from both control and treatment group.

Encouraged by our in vitro studies, we proceeded to assess the anti-proliferative effect of Tat-SP4 in vivo using a xenograft model. MDA-MB-231 cells were implanted subcutaneously on 6-week female nude mice and daily intraperitoneal injection (i.p.) of Tat-SP4 at 40 mg/kg started once tumor volume reached 100 mm^3^. Compared to the control group with daily injection of PBS, Tat-SP4 treatment reduced tumor volume by ~60% after 35 days (Fig. 7a, b). The tumor weight was reduced by ~50% as well (Fig. 7c). Additionally, Tat-SP4 treatment at 40 mg/kg showed no obvious toxicity to mice as measured by body weight and vital organs (Fig. 7d, e). Thus, Tat-SP4 potently inhibited tumor growth in an xenograft model of TNBC in vivo.

## Discussion

As an evolutionarily conserved endolysosomal recycling process, autophagy is essential for cell viability [11]. However, excessive autophagy has been reported to cause a unique form of cell death termed autosis [37]. This process has been observed in both cultured cells and tissues including neurons, cardiomyocytes and immune cells after severe stress like ischemia hypoxia or excessive autophagy induction by the Tat-Beclin 1 peptide [38–42]. The molecular mechanism of autosis is not well understand, particularly regarding how excessive autophagy leads to necrotic cell death. Autophagy machinery plays a prominent role in this process as genetic ablation of essential autophagy genes including Beclin 1, Atg13 and Atg14L all reduced autosis triggered by Tat-Beclin 1 [38]. Intriguingly, autosis also depends on Na^+^/K^+^ ATPase, as genetic ablation or pharmacological inhibition of this ion pump also blocked autosis [38]. Beclin 1 showed stronger co-immunoprecipitation with Na^+^/K^+^ ATPase in mice under autophagy- and autosis-inducing conditions such as starvation and ischemia–hypoxia [44]. Whether Beclin 1 directly interacts with Na^+^/K^+^ ATPase to modulate autosis still requires further biochemical and functional validation.

We have developed Beclin 1-targeting stapled peptides including Tat-SP4 that specifically bind to the Beclin 1 coiled coil domain to promote autophagy and enhance endolysosomal degradation [30, 31]. This mechanism is different from that for Tat-Beclin 1, which activates autophagy by releasing Beclin 1 from the negative autophagy regulator GAPR-1 [45]. Despite this difference, Tat-SP4 induced necrotic cell death in TNBC cells with prominent features of autosis, including continued adherence to substrate surface, rupture of plasma membrane and rescue only by digoxin, a cardioglycoside that blocks Na^+^/K^+^ ATPase, but not by inhibitors of other programmed cell death pathways like apoptosis, ferroptosis, and necroptosis. Our data validate Tat-SP4 as an autosis-inducing agent and suggests that this unique form of cell death may be induced by a broad collection of autophagy-inducing agents.

The autotic cell death induced by Tat-SP4 in TNBC cells does show three notable distinctions compared to that induced by Tat-Beclin 1 in HeLa and U2OS cells [38]. First of all, Tat-SP4 only induced mild increase of in autophagy as judged by the ~50% change in the level of LC3-II in TNBC cells. In contrast, Tat-Beclin 1 caused massive induction of autophagy with several fold of increase in the LC3-II level [38]. Yet Tat-SP4 and Tat-Beclin 1 showed comparable potency in terms of inducing autotic cell death with IC_50_ values of both peptides estimated at ~20 μM [38]. The lack of prominent autophagic response in TNBC cells is likely due to intrinsic autophagy deficiency caused by spontaneous monoalleilic deletion of Beclin 1 in breast cancer in general and low mRNA level of Beclin 1 in TNBC in particular [21, 24]. Thus autotic cell death can be triggered even with moderate increase in autophagy, as long as such change triggers downstream deleterious events such as dilated ER and electron-dense mitochondria [46].

Another notable feature of Tat-SP4 induced autotic cell death is the involvement of mitochondria in this process. Tat-SP4 caused prominent mitochondria dysfunction, as shown by loss of mitochondria membrane potential, increased ROS level and reduction in OXPHOS. These changes were detected in viable TNBC cells prior to autosis and live-cell imaging further confirmed widespread loss of the mitochondria membrane potential as the defining event prior to commencement of autotic cell death. In comparison, Tat-Beclin 1 only caused abnormal fragmentation of the mitochondria network in dying cells and together with ER abnormalities, which likely acts as a downstream event rather than cause of autosis [38]. Mitochondria play key roles in several cell death programs [36, 47]. Mitochondria outer membrane permeabilization (MOMP) is a signature event in apoptosis and leads to the release cytochrome C from inter-mitochondria membrane space to cytosol to activate caspases [36, 47]. Mitochondria also has extensive crosstalk with non-apoptotic cell death programs including necroptosis, ferroptosis, and pyroptosis [36, 47]. Furthermore, mitochondria have been reported to undergo permeability transition (MPT), leak pro-inflammatory molecules like mitochondrial DNA and precipitate necrotic cell death [36]. Our data has uncovered multiple aspects of mitochondria dysfunction in TNBC cells after Tat-SP4 treatment. Further studies are needed to identify the molecular targets involved in such dysfunction. It is possible excessive level of dysregulated autophagy may incur a unique form of mitochondria stress to induce autosis. The low mitochondrial OXPHOS activity observed in TNBC cells may serve as one of the biomarkers that define their vulnerability to autosis.

Lastly, our data investigating Tat-SP4 induced cell death adds to a growing body of evidence to demonstrate that autosis is a unique form of cell death triggered by excessive autophagy and without involvement of other cell death programs [48, 49]. On the other hand, factors that trigger autosis, such as ischemia hypoxia, can activate other cell death programs like apoptosis and necrosis as well. We reason cells may follow one or a few cell death pathways in context-dependent manner as driven by their susceptibility to each individual pathway. Susceptibility to autosis could be influenced by many factors, including endogenous autophagy capacity and mitochondria resilience as reported in this study. In this regard, intrinsic autophagy deficiency and quiescent mitochondria bioenergetics profile likely render TNBC cells particularly susceptible to autosis. Our designed peptides like Tat-SP4 may serve as potential therapeutic candidates against TNBC by targeting this vulnerability.

### Experimental section

#### Reagents and antibodies

Chloroquine (CQ; Sigma-Aldrich), epidermal growth factor (EGF; Gibco), EDTA-free protease inhibitor cocktail (Bimake), Trypsin (Invitrogen), Trypan Blue (Gibco), Z-VAD-FMK (Sigma-Aldrich), Necrostatin-1 (Nec-1; Sigma-Aldrich), Oligomycin (Cayman), FCCP (Cayman), Rotenone (Sigma-Aldrich), Antimycin A (Sigma-Aldrich), Matrigel (Corning), Erastin (Sigma-Aldrich), Ferristatin-1 (Fer-1; Sigma-Aldrich), Bafilomycin A1 (Baf A1; MedChemExpress), 3-Methyladenine (3-MA; MedChemExpress), anti-β-actin antibody (Santa Cruz Biotechnology; sc-47778; 1:2500 dilution), anti-LC3 antibody (Novus; NB100-2220; 1:1000 dilution), anti-p62 antibody (Abnova; H00008878-M01; 1:10,000 dilution), anti-EGFR antibody (Santa Cruz Biotechnology; sc-03; 1:2500 dilution), Anti-Mouse IgG-HRP (Sigma-Aldrich; A9044; 1:2500 dilution), Anti-Rabbit IgG-HRP (Sigma-Aldrich; A9169; 1:2500 dilution).

#### Chemical synthesis of stapled peptides

The Tat-SP4 stapled peptide was purchased from GL Biochem (Shanghai) Ltd. The synthesis process was the same as reported in our previous study [50]. Briefly, the peptide was synthesized by automated solid-phase method with olefin-containing amino acids incorporated at the designated positions. The hydrocarbon staple was formed on olefin-containing amino acids by ring-closing metathesis reaction using the Grubbs catalyst. Chemical structure and purity of the final product were characterized by HRMS and HPLC. Purity of each the peptide is >95%. Stock solution of the peptide was prepared by dissolving the sample in pure water to a concentration of 20 mM and stored at −20 °C.

#### Cell lines and cell culture

The triple negative breast cancer cell lines (MDA-MB-231, MDA-MB-468, BT549) were obtained from American Type Culture Collection (ATCC). They were all cultured in Roswell Park Memorial Institute (RPMI) 1640 Medium (Gibco) with the supplementation of 10% fetal bovine serum (FBS, Gibco). All the cell lines used in the experiments were monitored regularly by using MycoAlertTM PLUS Mycoplasma Detection Kit (Lonza) to avoid mycoplasma contamination.

#### Cell death assays

For IC50 measurement, cells were seeded into 96-well plates with the desired number (2.5 × 10^4^ cells/well for MDA-MB-231; 4 × 10^4^ cells/well for MDA-MB-468; 1 × 10^4^ cells/well for BT549). The cells were treated with different concentrations of Tat-SP4 when the confluent of attached cells reached 60–70%. At 24 h post-treatment, the number of viable cells was detected by Trypan Blue exclusion assay. The IC_50_ value was calculated from the curve fitted to concentration-response data sets. The experiments were repeated in triplicate. The IC_50_ value was also determined by measuring total ATP through CellTiter-Glo assay. The assay was performed using CellTiter-Glo Luminescent Cell Viability Kit according to the manufacturer’s instructions (Promega). For the 5-day growth curve, cells were seeded into a 24-well plate at 2.5 × 10^4^ cells/well for MDA-MB-231, 1 × 10^5^ cells/well for MDA-MB-468, and 3 × 10^3^ cells/well for BT549. Upon cell attachment, cells were treated with 10 or 20 μM Tat-SP4. Cells were allowed to grow up to 5 days post-treatment, and the viable cell number was checked every 24 h with Trypan Blue exclusion assay. The three TNBC cell lines were plated overnight in 96-well plates for cell death assays. Tat-SP4 was added together with apoptosis inhibitor, ferroptosis inhibitor, necroptosis inhibitor, or autosis inhibitor into the cells. After 24-h treatment, the cell viability was measured by trypan blue exclusion assay.

#### Immunoblot analysis

Cells were lysed in Laemmli sample buffer (62.5 mM Tris-HCl, pH 6.8, 2% SDS, 25% glycerol, 5% β-mercaptoethanol) with EDTA-free protease inhibitor cocktail (Bimake). Protein concentration was determined by the Bio-Rad Protein Assay (Bio-Rad). Proteins were separated by SDS-PAGE and transferred onto PVDF membrane (Millipore). The level of indicated proteins was blotted by incubating with primary antibody overnight at 4 °C and followed by secondary antibodies incubation for 1 h at room temperature. Protein bands were visualized by using ECL reagents. β-Actin was used as the loading control.

#### EGFR degradation assay

Cells were seeded in 6-well plates 1 day prior experiment. On the day of the experiment, cells were washed with PBS twice and serum-starved for 12 h. Endocytosis of EGFR was induced by treatment with 200 ng/ml of EGF only or supplement Tat-SP4 or 100 nM Baf A1 at 37 °C. Cells were collected at the indicated time after stimulation and lysed as described in immunoblot analysis.

#### Flow cytometry

For apoptosis, apoptosis was measured by flow cytometry following the standard protocol provided by the manufacturer (Thermo Fisher Scientific). Briefly, MDA-MB-231 cells in a 6-well plate were treated with indicated concentrations of Tat-SP4 for 24 h and were digested with trypsin. The harvested cells were washed twice with cold PBS and resuspended with 100 μl annexin-binding buffer containing 5 μl FITC annexin V (100 μg/ml) and 1 μl propidium iodide (100 μg/ml). The cells were incubated in the dark at room temperature for 15 min. The cell apoptosis was examined by the flow cytometer system with fluorescence emission at 530 nm and >575 nm (BD Accuri C6). For mitochondrial membrane potential, the change of ∆ψm is determined as the change of fluorescence intensity of tetramethylrhodamine methylester (TMRM). Cells grown in 6-well plates were treated with indicated concentrations of Tat-SP4 for 1 h. Then, the medium was removed, and the cells were stained with 100 nM TMRM for additional 30 min of incubation at 37 °C. After incubation, the cells were collected by trypsinization and subjected to a flow cytometer system with fluorescence emission at 570 nm. For ROS production, it is determined as the fluorescent intensity of the CellROX green probe (Thermo Fisher Scientific). Cells were treated with indicated concentrations of Tat-SP4 for 1 h, followed by trypsinization. The harvested cells were stained with CellROX reagent at a final of 500 nM for 1 h at 37 °C. After the incubation, cells were washed three times with cold PBS. The ROS generation was detected by flow cytometry with fluorescence emission at 530 nm. At least 30,000 events were collected for each sample for flow cytometry. The results were analyzed by BD Accuri C6 software.

#### Confocal microscopy imaging

MDA-MB-231 cells were stained with TMRM for 30 min at 37 °C. After incubation, live cell imaging was performed immediately once adding 20 μM Tat-SP4. The images were captured by Leica invert confocal microscope (TCS-SP8-MP system) with a ×63 oil immersion objective lens. During the imaging process, the cells were kept in the 5% CO_2_, 37 °C incubator.

#### Transmission electron microscopy

MDA-MB-231 cells were treated with 20 μM Tat-SP4 for 1 h. After treatment, the cells were harvested by trypsinization. The cell pellet was resuspended in PBS and immediately high pressure frozen (EM PACT2, Leica). The frozen cells were transferred under liquid nitrogen into an AFS freeze-substitution unit (Leica) containing anhydrous acetone with 0.4% uranyl acetate at −85 °C. After gradient infiltration with increasing concentration of HM20, samples were embedded and ultraviolet polymerized for ultra-thin sectioning and imaging. TEM examination was performed with a Hitachi H-7650 transmission electron microscope with a CCD camera (Hitachi High-Technologies) operating at 80 kV.

#### Seahorse analysis

Oxygen consumption of intact cells was measured using an XFe24 Extracellular Flux Analyzer (Agilent) following the standard protocol provided by the manufacturer. Briefly, one day before the experiment, MDA-MB-231 cells were seeded in an XF24 cell culture microplate with a density of 5 × 10^4^ cells/well. On the same day, 1 ml of XF calibrant was filled into each well of the utility plate to hydrate the sensor. The XF24 sensor cartridge was incubated in a non-CO_2_, 37 °C incubator overnight. On the day of the experiment, the cells were washed with PBS twice and incubated with XF assay medium containing 1 mM sodium pyruvate, 2 mM glutamine, and 10 mM glucose for 1 h in a non-CO_2_, 37 °C incubator. During the incubation, different compounds were loaded into reagent ports. In a typical Cell Mito Stress Test, the compounds were injected in order of 1 μM oligomycin (Port A), then 1 μM FCCP (Port B) and followed by 0.5 μM rotenone and 0.5 μM antimycin A (Port C). To test the efficacy of Tat-SP4, indicated concentrations of Tat-SP4 were loaded into Port A, and the other three compounds were loaded into Port B to D. The OCR was measured by XFe Analyzer (Agilent), and the data was analyzed by Wave software (Agilent) after cell number normalization. For measurement of respiratory activity in permeabilized cells, the main procedures were performed following essentially the protocol described in a previous publication [51]. Briefly, the procedures were the same as in typical Cell Mito Stress Test on 1 day prior to the experiment. On the day of the experiment, the cells were washed twice quickly with a nonionic mannitol plus a sucrose-based (MAS) buffer. Then, 500 μl MAS buffer was added to each well with the supplement of 4 mM ADP (Sigma). The compounds were injected into the cells with the sequential 1 nM plasma membrane permeabilizer (PMP) (Agilent), indicated concentrations of Tat-SP4, specific substrates and supplements for each complex (Complex I: 10 mM pyruvate and 1 mM malate; Complex II: 10 mM succinate and 2 μM rotenone; Complex III: 10 mM glycerol-3-phosphate and 2 μM rotenone; Complex IV: 10 mM ascorbate and 100 μM TMPD + 2 μM antimycin A), and followed by relevant inhibitors (Complex I: 2 μM rotenone; Complex II: 2 μM antimycin A; Complex III: 2 μM antimycin A; Complex IV: 20 mM azide). The OCR was measured by XFe Analyzer (Agilent), and the data was analyzed by Wave software (Agilent).

#### Animal study

Female athymic nude mice (nu/nu) (5–6 weeks old) were obtained from Beijing Vital River Laboratory Animal Technology Co., Ltd. for human xenografts. MDA-MB-231 cells were harvested and resuspended with PBS and Matrigel (Corning) in a ratio of 1:1 (v/v). 5 × 10^6^ cells were implanted subcutaneously with a 21 G needle. The mice were randomly grouped into the control group and Tat-SP4 treatment group (*n* = 7) once the tumor volume reached 100 mm^3^. To minimize the potential imbalance factors that may influence the result of the experiment, the randomization followed the rules that the body weight and starting tumor volume of the mice in the two groups were quite close. Tumor volume was measured by caliper and calculated by the equation of length × width^2^ × 0.5. The administration of Tat-SP4 was performed via daily intraperitoneal (i.p.) injection at 40 mg/kg. PBS, as a vehicle of Tat-SP4, was injected for the control group. The tumor volume and body weight were recorded every 3 days until the day of tumor harvest. The animal studies were conducted in accordance with the protocol approved by the Animal Subjects Ethics Subcommittee of The Hong Kong Polytechnic University and The Hong Kong Polytechnic University Shenzhen Research Institute. There is no blinding in this experiment.

#### Statistics

All the statistical analyses were performed by GraphPad Prism 8.0 (GraphPad Software). The results of western blots were quantified by ImageJ software (NIH, USA). For comparisons between two groups, unpaired two-tailed Student’s *t* tests were used. For comparisons between three or more groups, one-way ANOVA with Dunnett’s multiple comparisons test were performed to determine the significance. Results were presented as mean ± SEM. All statistical analyses were two-sided, and *P* values < 0.05 were considered to be statistically significant.

### Supplementary information

Western blot raw data

Video S1

## Data Availability

All data are available in the main text or the Supplementary Materials.
