# Supplementary material for: An autophagy-inducing stapled peptide induces mitochondria dysfunction and triggers autotic cell death in triple-negative breast cancer
Source: Cell Death Discov. 2023 Aug 19;9:303. doi: 10.1038/s41420-023-01600-0 (PMC10439894; doi:10.1038/s41420-023-01600-0)

Figure 1b,c

MDA-MB-231
Repeat 1

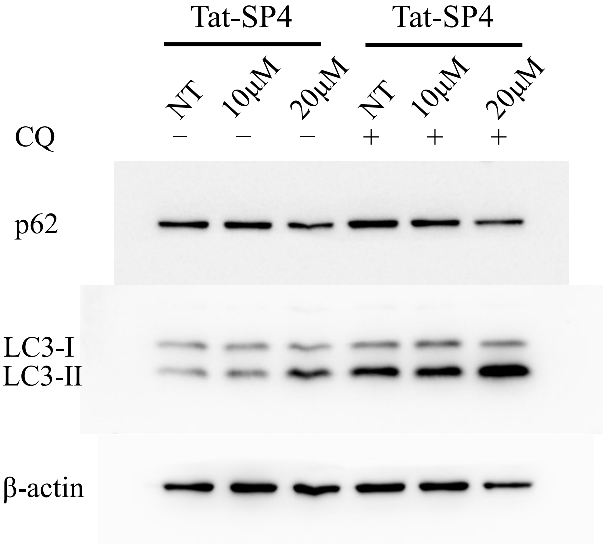

MDA-MB-231
Repeat 2

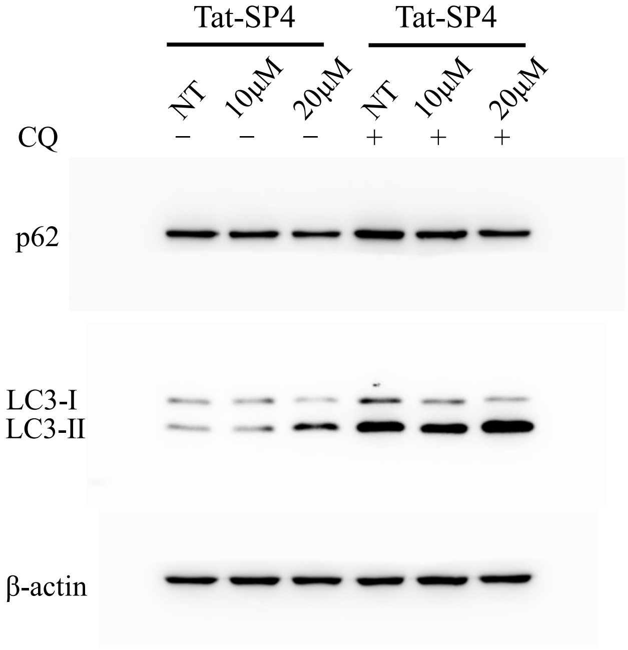

MDA-MB-231
Repeat 3

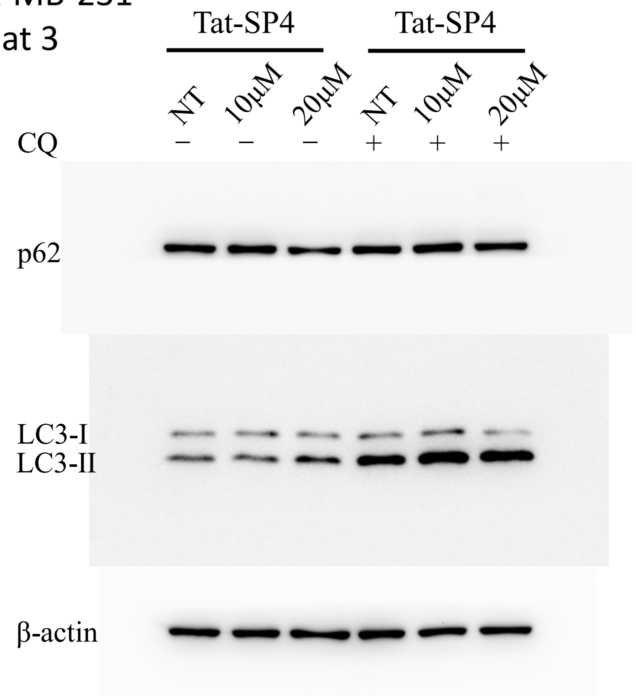

MDA-MB-231
Repeat 4

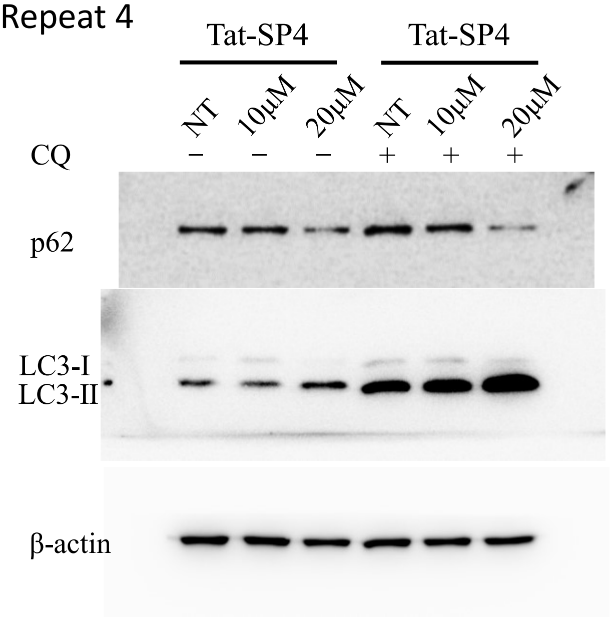

Figure 1d,e

MDA-MB-468
Repeat 1

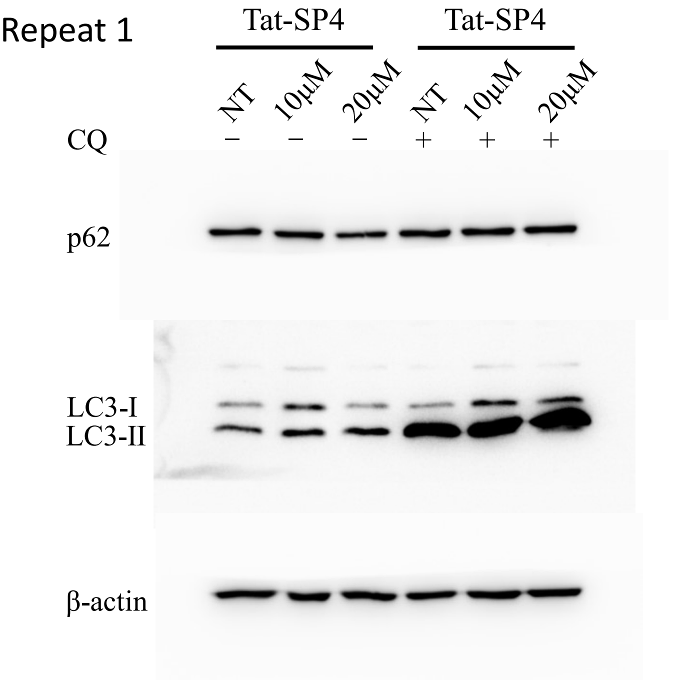

MDA-MB-468
Repeat 2

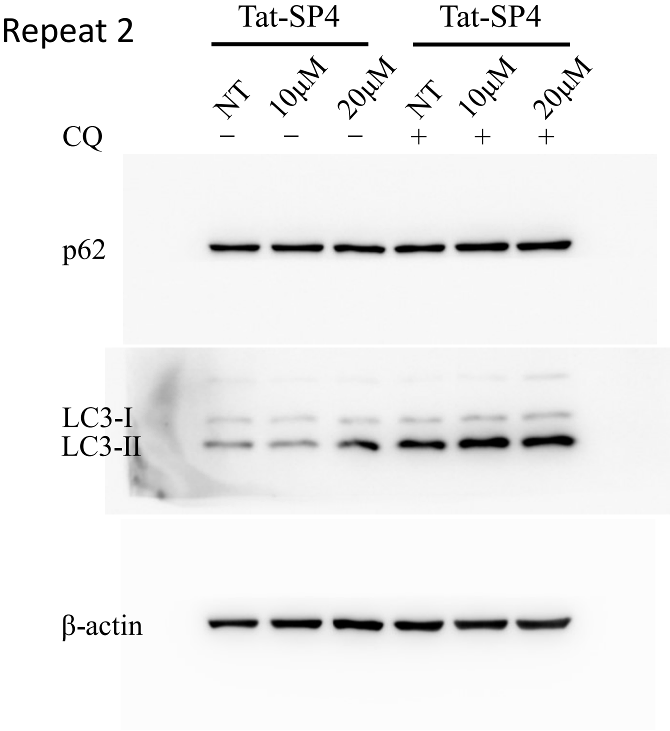

MDA-MB-468
Repeat 3

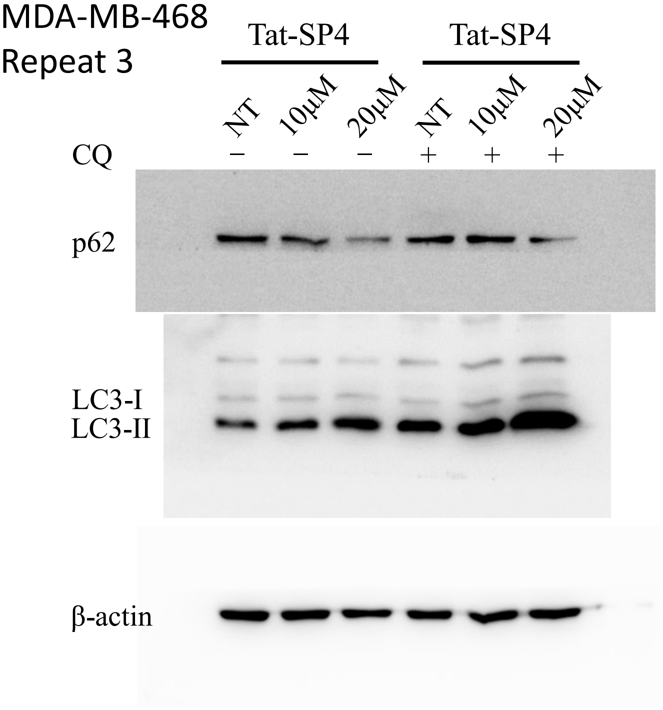

MDA-MB-468
Repeat 4

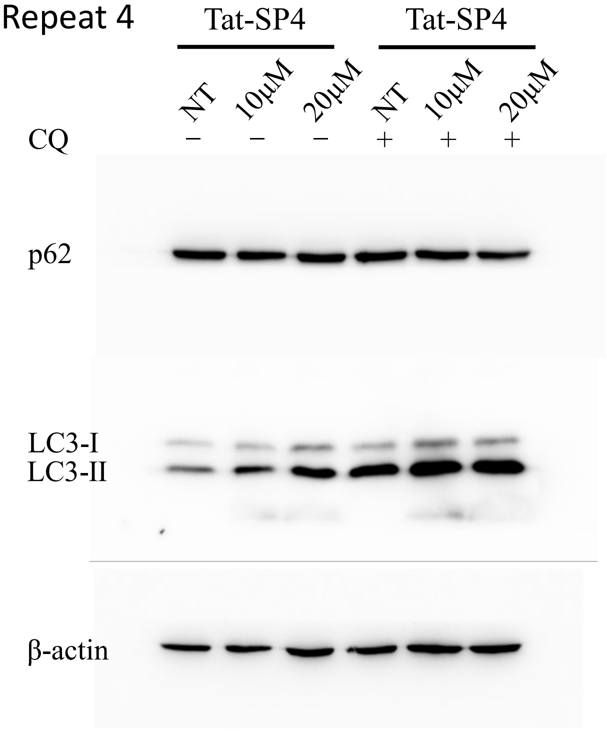

Figure 1f, g

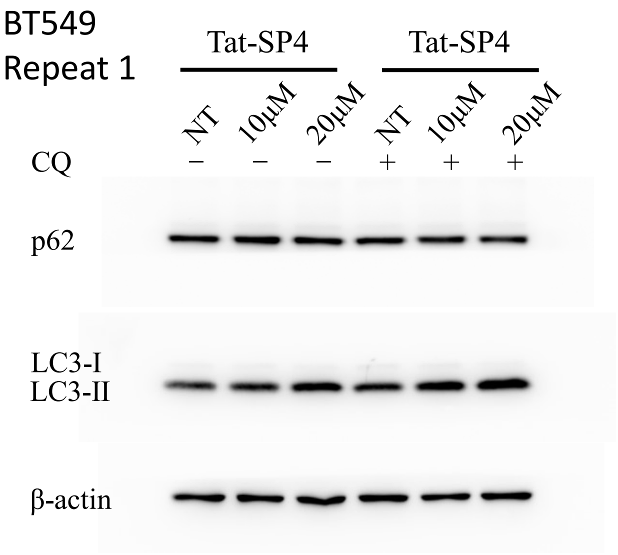
BT549
Repeat 1
Tat-SP4
Tat-SP4
NT
10μM
20μM
NT
10μM
20μM
CQ
−
−
−
+
+
+
p62
LC3-I
LC3-II
β-actin

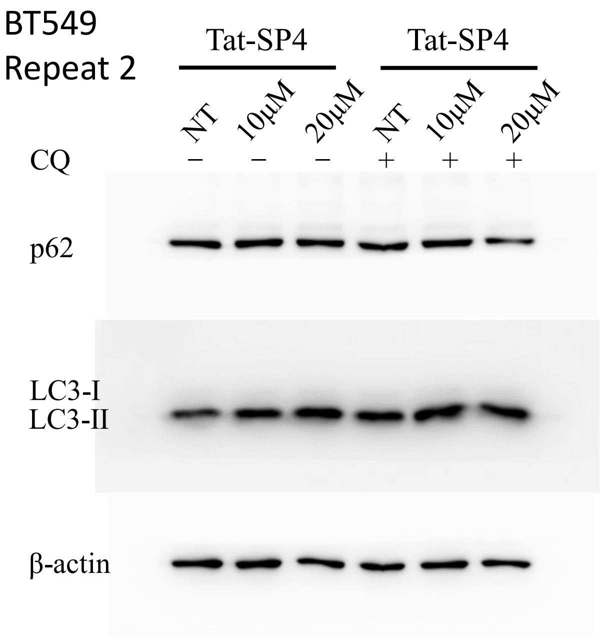
BT549
Repeat 2
Tat-SP4
Tat-SP4
NT
10μM
20μM
NT
10μM
20μM
CQ
−
−
−
+
+
+
p62
LC3-I
LC3-II
β-actin

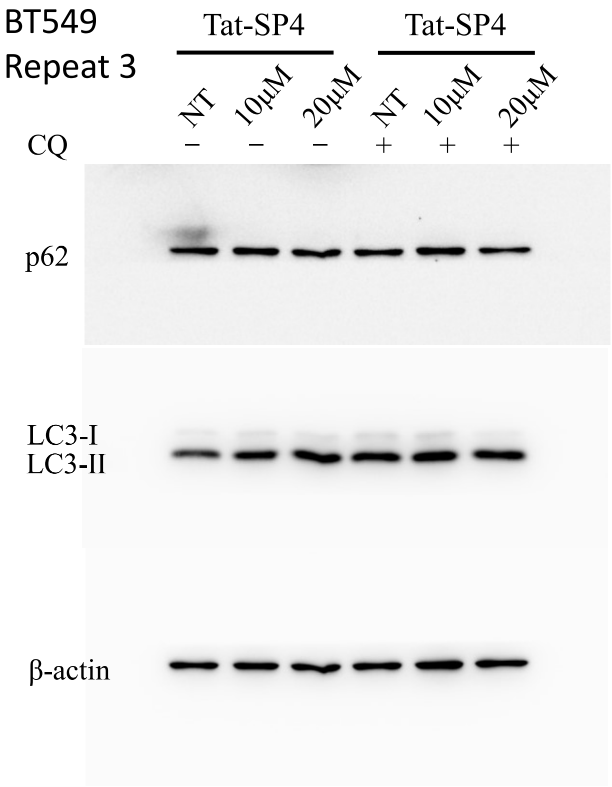
BT549
Repeat 3
Tat-SP4
Tat-SP4
NT
10μM
20μM
NT
10μM
20μM
CQ
−
−
−
+
+
+
p62
LC3-I
LC3-II
β-actin

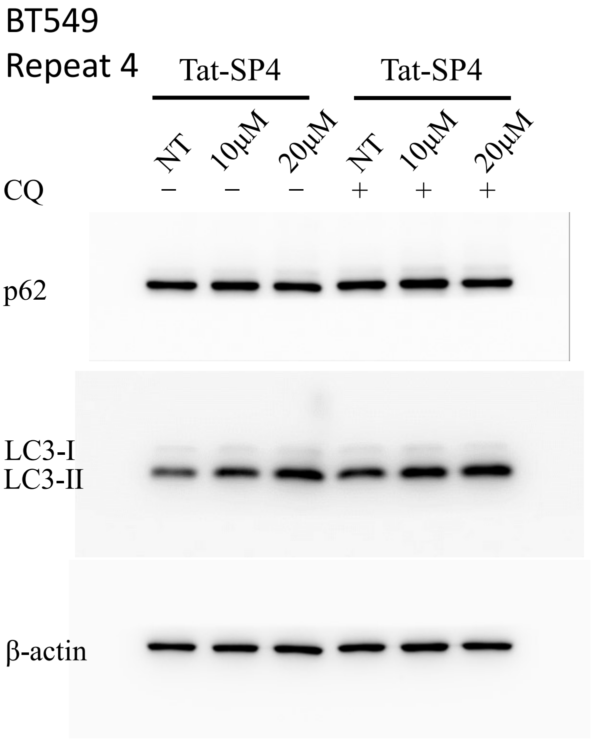
BT549
Repeat 4
Tat-SP4
Tat-SP4
NT
10μM
20μM
NT
10μM
20μM
CQ
−
−
−
+
+
+
p62
LC3-I
LC3-II
β-actin

Figure 1h

MDA-MB-231
Repeat 1

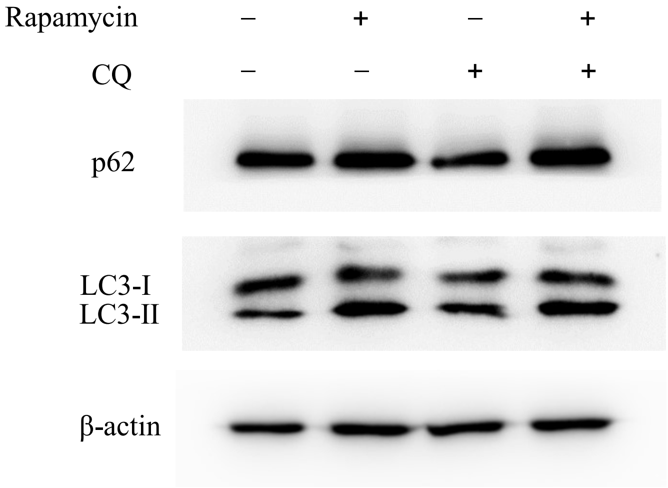

MDA-MB-231
Repeat 2

MDA-MB-231
Repeat 3

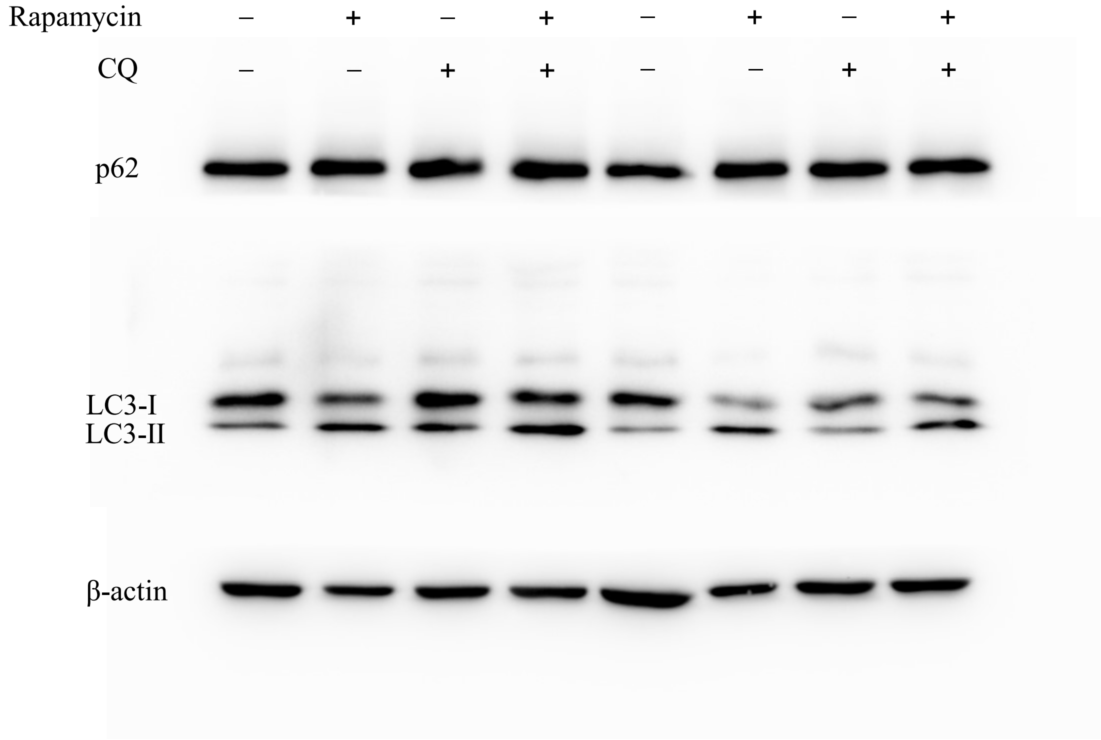

MDA-MB-231
Repeat 4

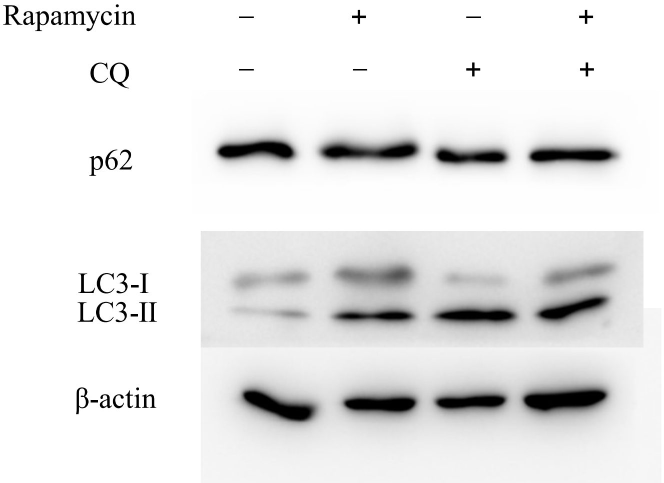

Figure 1i

MDA-MB-468
Repeat 1

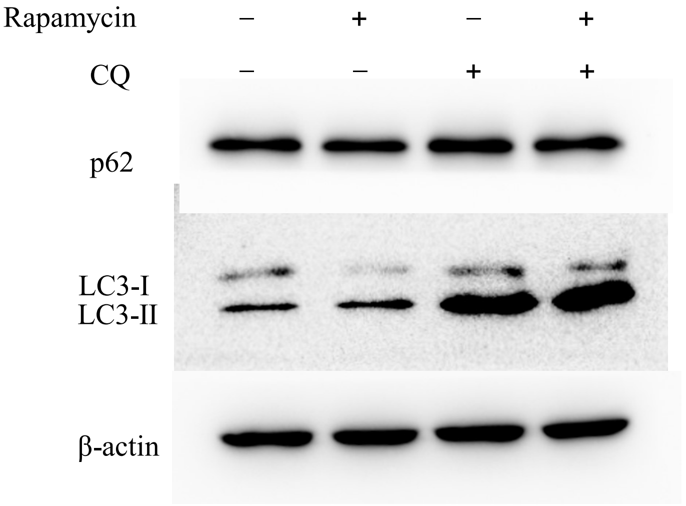

MDA-MB-468
Repeat 2

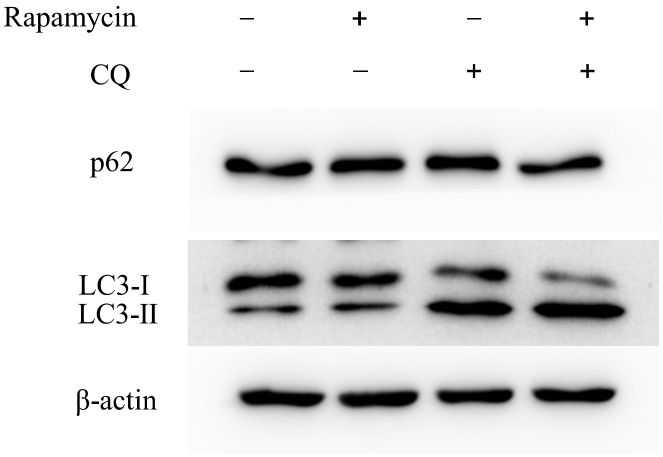

MDA-MB-468
Repeat 3

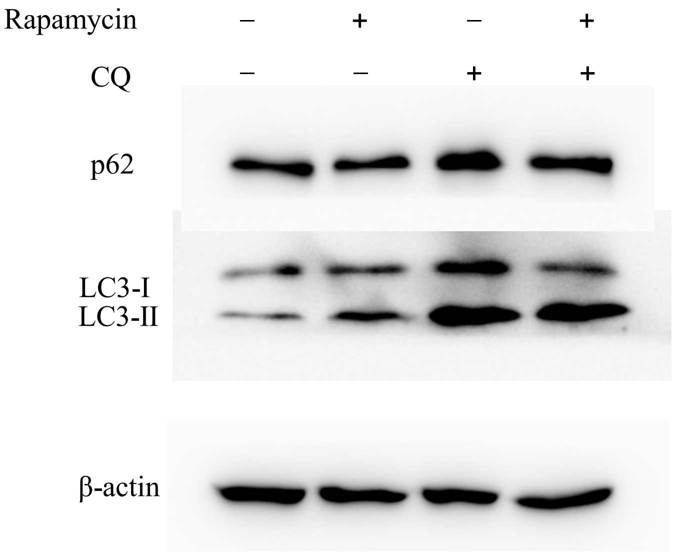

MDA-MB-468
Repeat 4

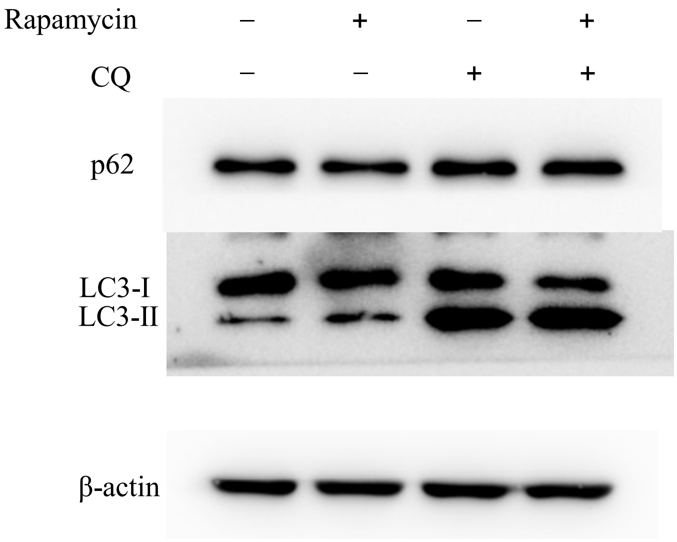

Figure 1j

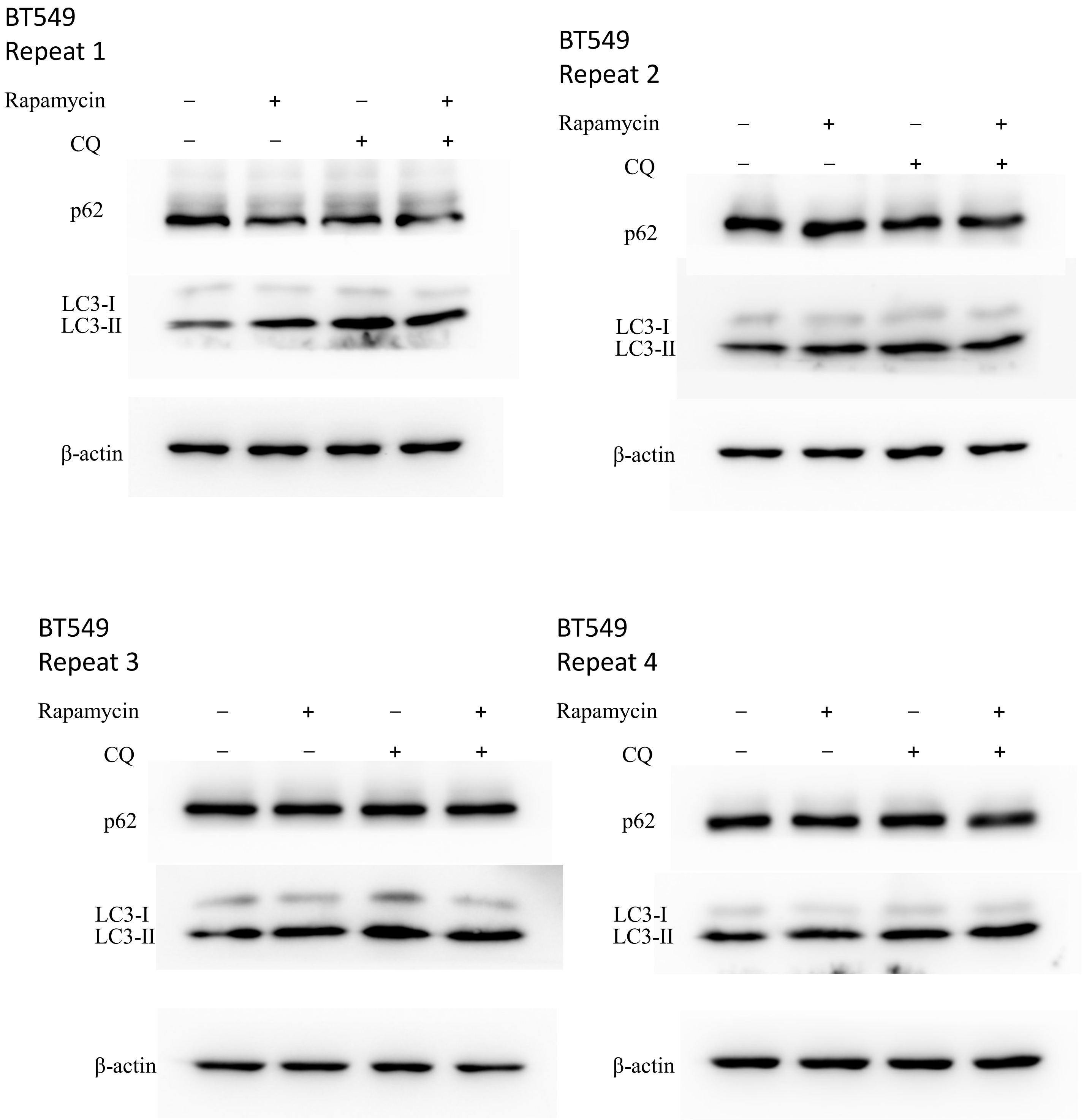
BT549
Repeat 1
Rapamycin – + – +
CQ – – + +
p62
LC3-I
LC3-II
β-actin
BT549
Repeat 2
Rapamycin – + – +
CQ – – + +
p62
LC3-I
LC3-II
β-actin
BT549
Repeat 3
Rapamycin – + – +
CQ – – + +
p62
LC3-I
LC3-II
β-actin
BT549
Repeat 4
Rapamycin – + – +
CQ – – + +
p62
LC3-I
LC3-II
β-actin

Figure 2a

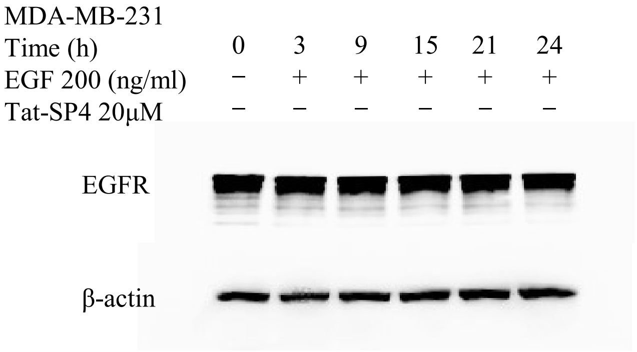

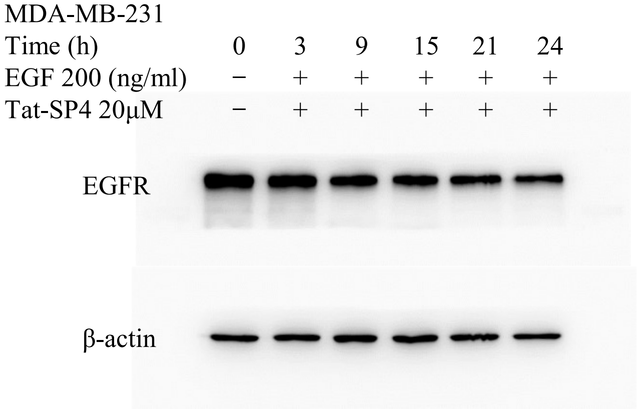

Figure 2b

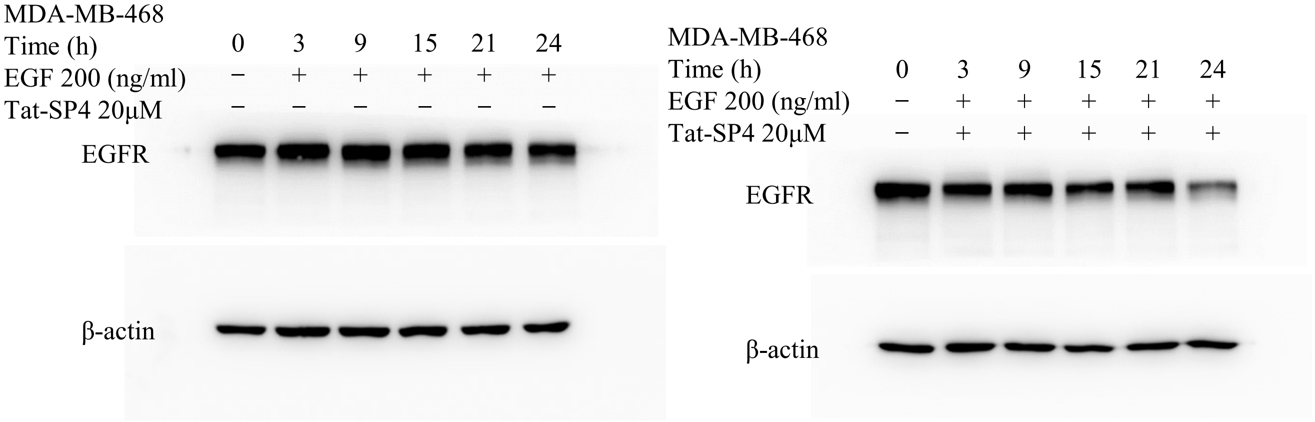

Figure 2c

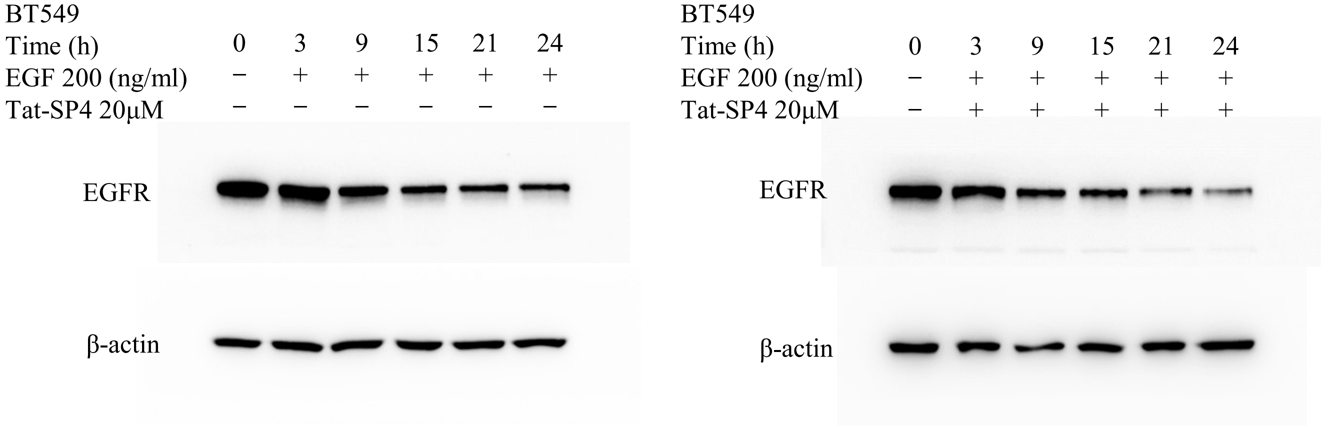

Figure 2d

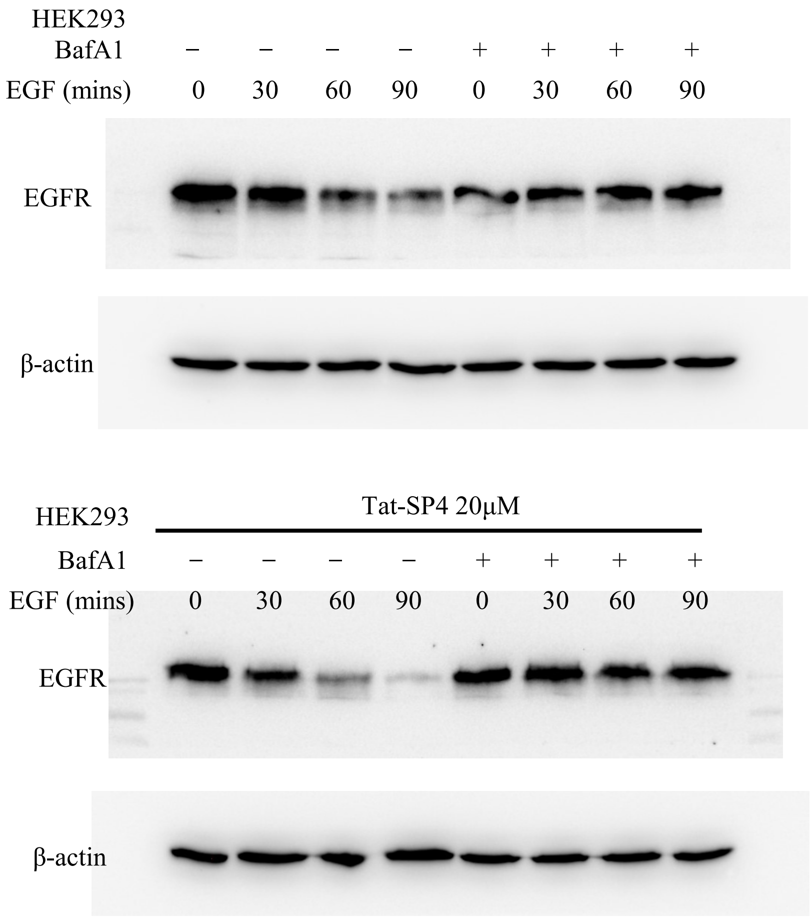

Figure 2e

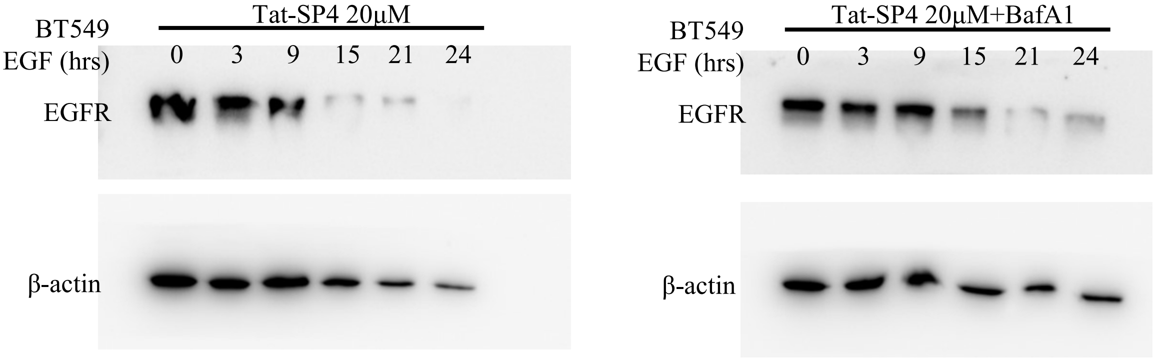

Supplement: Supplementary file 1 — Western blot raw data [file 41420_2023_1600_MOESM1_ESM.pdf]
